# Degradable fibrin hydrogels for transplantation of iPSC-derived retinal pigment epithelial cell monolayers

**DOI:** 10.3389/fcell.2025.1739620

**Published:** 2026-01-14

**Authors:** Alan D. Marmorstein, Brittni A. Scruggs, Travis Knudsen, Matthew Hill, Francesca N. Kopp, Emma Trncic, David Korda, Evan Atherton, Aubrey Berger, Silvia C. Finnemann, Jarel Gandhi, Raymond Iezzi

**Affiliations:** 1 Retinal Regenerative Medicine Laboratory, Department of Ophthalmology, Mayo Clinic, Rochester, MN, United States; 2 Department of Pediatrics, Mayo Clinic, Rochester, MN, United States; 3 Biomedical Graduate Program, Mayo Clinic, Rochester, MN, United States; 4 Department of Biological Sciences, Fordham University, Bronx, NY, United States

**Keywords:** fibrin, geographic atrophy, iPSCs, retinal pigment epithelium, scaffold, stem cells

## Abstract

Death or dysfunction of retinal pigment epithelium (RPE) cells occurs in age-related macular degeneration (AMD) and certain inherited retinal dystrophies (IRDs). Induced-pluripotent stem cell (iPSC) derived-RPE have been used in early-stage clinical trials to treat AMD and IRDs by injecting them as a cell suspension or monolayers. While RPE transplant shows therapeutic potential, issues ranging from failure to repopulate the entire treatment area, clumping and monolayer folding, and a foreign body response to the support have been reported. We’ve shown that RPE can be grown on high concentration (>30 mg/mL) degradable fibrin hydrogels, and that cell free fibrin hydrogels implanted in the subretinal space degrade without causing inflammation. Here we describe manufacture and surgical implantation of degradable fibrin hydrogels carrying iPSC-RPE into a porcine model of geographic atrophy (GA). Large (15.25 × 58.42 × 0.2 mm) fibrin gel blanks were produced by injection molding, and iPSC-RPE were grown on their surface. Using a mechanical punch, the blank was subdivided into 1.5 × 5.0 × 0.2 mm doses, which fit a custom tool used for storage and surgical placement. Following aseptic packaging, RPE and gels were stable at 37 °C for at least 7 weeks. When transplanted into a pig model of GA, the fibrin scaffold degraded in <1 month and the iPSC-RPE provided partial rescue from GA as assessed by preservation of photoreceptors and blood flow in the choriocapillaris. We conclude that iPSC-RPE delivered on degradable fibrin hydrogels represent a potentially safe and effective approach to RPE transplantation.

## 1 Introduction

Death or dysfunction of retinal pigment epithelial (RPE) cells occurs in age-related macular degeneration (AMD) (Fleckenstein et al., 2018) and a subset of inherited retinal diseases (IRDs) (Grenell et al., 2024; Johnson et al., 2017; Marmorstein et al., 2007; Ng et al., 2024). Since the first transplant of RPE cells into RCS rats (Li and Turner, 1988) was observed to overcome RPE dysfunction and rescue photoreceptors, RPE transplant has been considered a potential therapy for AMD and IRDs (da Cruz et al., 2007; Zarbin et al., 2019). As early as the 1990s, it was recognized that RPE could be transplanted as either a cell suspension or a monolayer (Algvere et al., 1994; Algvere et al., 1997; Algvere et al., 1999). Cell suspensions are simpler in pre-operative preparation and surgical approach, however, they do not completely repopulate the target area of the transplant (Schwartz et al., 2015; Schwartz et al., 2016). Despite this, there is growing evidence for efficacy of cell suspensions in human clinical trials (Algvere et al., 1997; Algvere et al., 1999; Schwartz et al., 2015; Schwartz et al., 2016; Rao et al., 2025; Sugita et al., 2020; Ho et al., 2022) and stem cell derived RPE cell suspensions continue to be investigated [e.g. NCT06394232 (Soundararajan et al., 2025), NCT04627428 (Rao et al., 2025), NCT02286089 & NCT05626114 (Ho et al., 2022), NCT06394232 (Soundararajan et al., 2025), and NCT03178149].

The RPE is a simple cuboidal epithelium that separates the choroid from the neurosensory retina. Among its many functions, the RPE maintains the environment of the subretinal space, phagocytoses shed photoreceptor outer segments, and regulates fluid and nutrient transport between the blood supply in the choroid and the photoreceptors (Marmorstein, 2001; Fields et al., 2020; Strauss, 2005). The breaking down of a RPE monolayer into a cell suspension disrupts cell polarity, cell-cell junctions, and other phenotypic properties that facilitate their function. To preserve these phenotypic characteristics, numerous studies have examined transplantation of RPE as a monolayer (Algvere et al., 1994; Algvere et al., 1997; Schwartz et al., 2015; da Cruz et al., 2018; Mandai et al., 2017; Sharma et al., 2019). In its simplest form, RPE monolayers have been delivered unsupported (Algvere et al., 1994; Algvere et al., 1997; Mandai et al., 2017). In the first clinical trial of an induced pluripotent stem cell (iPSC) derived cell therapy Mandai et al. (2017) transplanted unsupported RPE monolayers. While there is some evidence of efficacy in RPE monolayer transplants (da Cruz et al., 2018; Mandai et al., 2017; Kashani et al., 2021; Kashani et al., 2018) and perhaps a lower incidence of rejection for allogeneic monolayers *versus* cell suspensions (Algvere et al., 1994; Algvere et al., 1997), the use of unsupported monolayers can result in clumping and folding of the transplanted RPE cells, and allows for minimal control of polarity upon surgical insertion (Algvere et al., 1999; Mandai et al., 2017; Takagi et al., 2019; Soroushzadeh et al., 2022). These problems can be solved by use of a scaffold to support the monolayer, prevent its folding, and preserve its polarity during surgical insertion.

A variety of materials have been proposed for use as scaffolds for RPE transplant. Supports composed of polyethylene terephthalate (PET) or parylene have both been used in phase 1/2a clinical studies to treat AMD (da Cruz et al., 2018; Kashani et al., 2018). Those studies have demonstrated that this approach allows delivery of an intact monolayer that covers the target area. However, both materials are non-degradable and persist in the eye. Five-year follow-up on individuals receiving embryonic stem cell (ESC) derived RPE on PET scaffolds found reduced persistence of transplanted RPE and fibrosis consistent with a foreign body response (Soomro et al., 2024). Follow-up of recipients of ESC-derived RPE on parylene scaffolds demonstrated the presence of inflammatory cells adjacent to the transplant 2 years postoperatively and loss of transplanted cells or changes in cell pigmentation (Kashani et al., 2021; Humayun et al., 2024). The persistence of non-degradable scaffolds prevented assessment of any effect on the choroid, and persistence of the scaffold may cause adverse effects, including hemorrhage and edema (Kashani et al., 2021; Soomro et al., 2024; Humayun et al., 2024). In these studies there was some indication of efficacy as measured by improvement or stabilization of best corrected visual acuity in a subset of transplant recipients (da Cruz et al., 2018; Kashani et al., 2021; Kashani et al., 2018; Soomro et al., 2024; Humayun et al., 2024); however, the unsupported monolayer transplants (Mandai et al., 2017) appeared to exhibit long term RPE survival as well and could be demonstrated to support both photoreceptors and choroid up to 4 years after transplantation (Mandai et al., 2017; Takagi et al., 2019).

Iti is not yet clear whether retention of the mature RPE phenotype *at the time* of transplantation is critical to the success of the procedure. If it is, however, then a more compliant degradable scaffold could be advantageous. Hotaling et al. (2016) reported on use of poly(lactic-*co*-glycolic) acid (PLGA) to produce a degradable scaffold for RPE transplant. PLGA scaffolds have been used in animal studies (Sharma et al., 2019) and are currently in phase 1 trials of autologous iPSC-derived RPE for the treatment of AMD [NCT04339764 (Hutton, 2022)]. PLGA supports are promising but also have their potential drawbacks. PLGA can be toxic (Stromberg et al., 2023; Chiu et al., 2021) and may elicit a foreign body response in the eye (Thackaberry et al., 2017). Furthermore, the support reportedly begins to degrade upon placement in culture medium thus limiting their storage shelf-life (Sharma et al., 2019; Hotaling et al., 2016). Popelka et al. (2015) developed a degradable scaffold from poly(L-lactide-co-DL-lactide; PDLLA). PDLLA membranes have a longer half-life (5–6 months) and Lytvynchuk et al. (2022) have demonstrated proof-of-concept for RPE transplantation on PDLLA scaffolds in Yorkshire minipigs. Other materials ranging from silk fibroin, alginate, hyaluronic acid, and various combinations of these and other materials have all been suggested for use as scaffold for RPE transplantation (Mazumder et al., 2012; Shamsnajafabadi et al., 2022; Suzuki et al., 2019; Thomas et al., 2021; Wei et al., 2022). While the majority of the later materials have been validated in rodents, silk fibroin and alginate are not readily degraded *in vivo* and none have been tested in large animal models that more closely mimic human eyes and surgical procedures.

We have focused on developing high concentration (>30 mg/mL) fibrin hydrogels as a support for RPE monolayers (Gandhi et al., 2019; Gandhi et al., 2020; Gandhi et al., 2018). Fibrin is a cross-linked fibrillar network formed spontaneously after the activation of fibrinogen by the enzyme thrombin. Fibrin forms the scaffold for blood clots and has a well-established cascade of activation, formation, degradation, and clearance. The enzymes catalyzing these actions are all found naturally in the blood, and many have been commercialized for medical use (Undas and Ariens, 2011). Fibrin tissue glues, for example, have been in use for decades with an excellent safety record (Spotnitz, 2014). We have shown that cell free fibrin hydrogels degrade within 4–8 weeks when placed in the subretinal space of the pig eye (Gandhi et al., 2020), and <1 week when placed on the epiretinal surface of the pig eye or <12 days when placed on the epiretinal surface of a human eye (Scruggs et al., 2025). In all of these cases without promoting an immune response (Gandhi et al., 2020; Scruggs et al., 2025).

In our prior work, gels were formed by pressing, which resulted in a significant variation in gel thickness and surface topology due to the rapid polymerization of fibrin hydrogels at supraphysiologic concentrations (Gandhi et al., 2020; Gandhi et al., 2018). By applying our observation that the initial gelation of fibrin can be slowed with the azo-dye trypan blue (Gandhi et al., 2021), we have developed an injection molding process for the manufacture of large, 3-dimensionally shaped, high concentration fibrin hydrogels (Scruggs et al., 2025). Here we report on modification of that method to permit RPE monolayer culture on the hydrogel blanks. We also describe an integrated storage/inserter system for the transplantable iPSC-RPE/fibrin gel and its placement in the subretinal space of a pig model of advanced AMD that we recently developed (Iezzi et al., 2024). Lastly, we demonstrate that the fibrin hydrogel degrades rapidly, leaving behind an RPE monolayer that exerts a rescue effect. Based on these data, we conclude that iPSC-derived RPE delivered on degradable fibrin hydrogel scaffolds represent a potentially safe and effective approach to RPE transplantation for treatment of macular degeneration.

## 2 Results

### 2.1 Fabrication of fibrin hydrogel blanks

In our previous work with high concentration fibrin hydrogels, they were formed by pressing (Gandhi et al., 2019; Gandhi et al., 2020; Gandhi et al., 2018). During the course of our studies, we found that the thickness of pressed gels was highly variable. This was in part due to the rapid initial gelation of fibrin at supra-physiological concentrations. We later found that the initial gelation could be slowed by the addition of select azo-dyes such as trypan blue and Evans blue (Gandhi et al., 2021). Trypan blue is used in vitreoretinal surgery to stain epiretinal membranes and the inner limiting membrane of the retina, so we choose to add trypan blue to our gelation mix. Doing so slowed gelation kinetics enough to allow us to use injection molding rather than pressing to fabricate a larger hydrogel with control over all three dimensions.

To use injection molding, we needed to design a mold that would yield a suitable blank. Minimum criteria for the blank were that it could yield >60 oval shaped doses of 1.5 × 5.0 × 0.2 mm and be in a format that could be utilized in cell culture for >30 days prior to punching/cutting of individual doses. This requires that the hydrogel have mechanical support for >30 days and that the hydrogel blank and support fit in a suitable cell culture vessel. To meet these criteria, it was considered essential, upon opening, that the gel blank remained adhered to the mold cavity, that the mold cavity be on only one side of the mold, that the mold plate not float in aqueous solutions, and that the mechanical support be produced from a material that is biocompatible and could meet ISO 10993 specifications.

**FIGURE 1:**
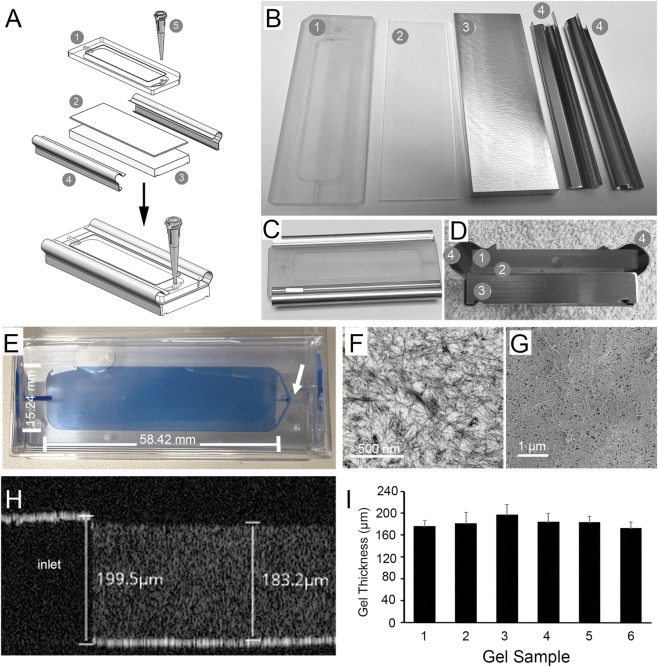
Gels were fabricated by injection molding. A schematic representation of the mold is shown in **(A)** along with a breakdown of the individual components in **(B)**. Molds are composed of a top plate (1), gasket (2), bottom plate (3), and two clips (4), which are used to hold the mold parts together. A disposable tapered inlet and fitting (5) is used to fill the mold. Panel **(C)** shows a top view of a mold assembly, and a side view is shown in **(D)** with each component labeled as in B. The mold is filled via a tapered inlet (white arrow in E). Following filling the gel is cured and molds are disassembled with gels remaining in the top plate cavity and stored in PBS **(E)**. Note the channels running from the inlet (arrow in E) that permit even filling of the mold. TEM demonstrates that gels are composed of a dense array of fibrin microfibrils **(F)**. The surface of the gel is smooth and contains crater-like voids when examined using SEM **(G)**. OCT imaging of a gel while still supported in the mold cavity of a top plate **(H)** shows that the mold cavity has a depth of _~_200 m, and the gel has a measured thickness of _~_183 m. The average ± SD of gel thickness from measurements made at 66 points in each of six different gels fabricated by two different operators is shown in **(I)**.

Based on extensive prototyping, we settled on a single use disposable mold design (Figures 1A–D) that uses a machined polycarbonate top plate with an 18-gauge tapered inlet port placed perpendicular to the mold. The polycarbonate plate is clear, permitting the operator to observe its filling during fabrication, and unlike polystyrene, the polycarbonate plate does not float in aqueous solution. A set of channels were milled in the mold cavity running from the inlet port to the middle and both outside edges to facilitate even filling (Figures 1B,C,E). The top plate is separated from a milled aluminum bottom plate *via* a 1/32″ sheet of silicone (Figures 1A,B,D), which serves as a gasket to seal the mold, maintaining pressure during injection, preventing leakage of the gelation mix, and preventing formation of air pockets. The pieces are held in place by clips placed on the long side of the mold (Figures 1A,C).

Fibrin gel fabrication was as described in Scruggs et al. (2025). Following curing at 37 °C, the process departs from that used in our prior work (Scruggs et al., 2025) as molds are opened and the gels hydrated in sterile PBS containing 2.5 mg/mL TXA and stored sterile at 4 °C (Figure 1E). At this point, the gels are blue in color due to the presence of trypan blue. Examination of gels using TEM showed that the gels have a fibrous structure similar to that observed previously (Gandhi et al., 2018; Scruggs et al., 2025; Gandhi et al., 2021) (Figure 1F), while SEM showed the surface to be relatively smooth with fibrils aligned parallel to the top surface plane interdigitated with crater-like voids across the surface (Gandhi et al., 2018; Scruggs et al., 2025; Gandhi et al., 2021) (Figure 1G). OCT measurements showed that gels were generally uniform in thickness averaging 182.9 ± 3.4 µm (mean ± SE, N = 6) with individual blanks varying in thickness by ± 11–20.3 µm with an average variance of 7.93% (Figures 1H,I).

### 2.2 Mechanical characteristics of fibrin hydrogel blanks

**FIGURE 2:**
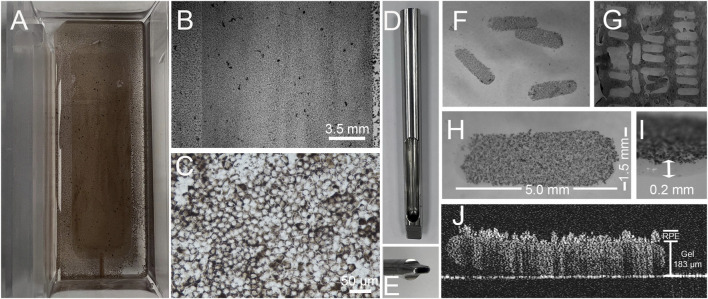
Growth of iPSC-RPE on fibrin hydrogels. Gels supported by top plates fit snuggly in the chamber of a four-well tissue culture dish **(A)**. RPE grown on the hydrogel densely cover the fibrin gel but adhere loosely to the polycarbonate side rails of the top plate surrounding the mold cavity. A higher magnification view shown in **(B)** illustrates the difference in cell density on the fibrin gel vs the polycarbonate sides of the top plate. Examination of cells on the fibrin gel at higher magnification **(C)** shows them to be a continuous “cobblestone” layer of cells. Using a custom punch **(D)**, which is designed to form ovals of 1.5 × 5.0 mm **(E)**, individual “doses” **(F)**, can be punched from the gel blank **(G)**. A top-down view **(H)** shows RPE on the top surface of the gel. An oblique view of a dose **(I)** shows that the cells do not penetrate the fibrin gel. OCT imaging **(J)** shows RPE sitting on the top surface of a dose which has exhibited no change in gel thickness due to growth of RPE cells.

For our purposes, the fibrin hydrogels must possess sufficient stiffness and elasticity to be handled and loaded in a surgical inserter instrument while being compliant enough not to damage surrounding tissues during placement. Young’s modulus for freshly fabricated gels was determined to be 0.053 ± 0.01 MPa, similar to our previously reported findings (Gandhi et al., 2018; Scruggs et al., 2025; Gandhi et al., 2021) and close to that of the retina, which is ∼0.02 MPa (Ferrara et al., 2021). Gels had sufficient stiffness and rigidity to utilize punching as a means to create smaller individual doses (Figures 2D–G).

A critical property of the gel is that it must be degradable, yet, not degrade during cell culture and storage. We have previously shown that gels produced using this or similar formulations are degradable (Gandhi et al., 2020; Gandhi et al., 2018; Scruggs et al., 2025; Gandhi et al., 2021); however, we have also shown that RPE cells grown on the fibrin will degrade the gel in the absence of aprotinin, a 6.5 kDa polypeptide protease inhibitor (Gandhi et al., 2019; Gandhi et al., 2018). Aprotinin is a part of the Tisseel kit, but we eliminated it because we found that it significantly impeded degradation of the gel and did not effectively wash out of the gel when used to stabilize it during cell culture (Gandhi et al., 2018; Scruggs et al., 2025). To address this, we substituted TXA a synthetic analog of lysine with a mass of 157 Da. Clinically, both are used to prevent bleeding; however, TXA is considered safer than aprotinin and is available over the counter in some countries. As shown in Figures 2I,J, culture of RPE cells on fibrin gels in media containing TXA results in a retention of the thickness of the gel.

### 2.3 Growth of RPE on fibrin hydrogels

**FIGURE 3:**
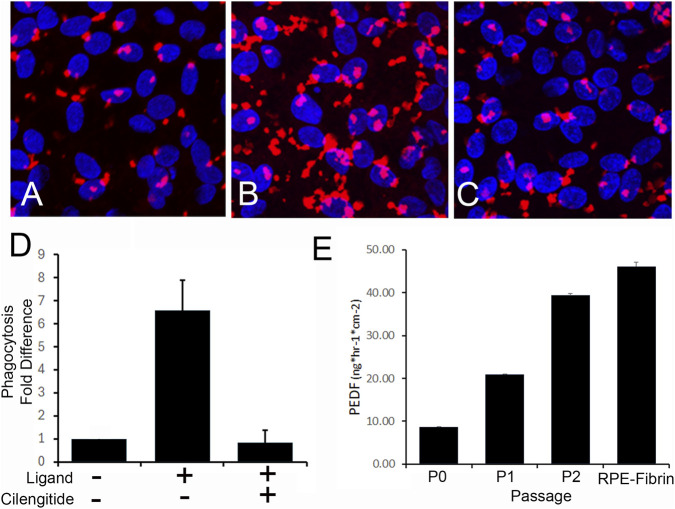
Functional characterization of iPSC-RPE. **(A)** Phagocytosis of iPSC-derived RPE was assessed by challenge with POS (RED in **A–C**) alone, or POS in the presence of RPE phagocytic receptor ligands MFG-E8 and Protein-S **(B,C)** without **(B)** or with **(C)** cilengitide integrin inhibitor peptide. Nuclei (Blue) in A-C were stained with DAPI. **(D)** A >6-fold increase in POS uptake was induced by the presence of RPE receptor ligands (*p* < 0.0001). This increase in phagocytosis was effectively competed by cilengitide. **(E)** PEDF, as a potency marker, was assessed in the medium of iPSC-RPE cultures following 1 month of differentiation at different passages using an ELISA assay. The rate of PEDF accumulation in the medium increased with each passage. Data in **(D)** are mean ± SD (*n* = 4). Data in **E** are mean ± SD (*n* = 3).

For these studies, we used RPE cells derived from the iPSC line 22/1. RPE differentiated from 22/1 formed highly pigmented monolayers of cells (Figure 2) with a cobblestone appearance (Figures 2C,H). The cells exhibited integrin and MERTK-receptor dependent phagocytosis (Finnemann et al., 1997a) of photoreceptor outer segment fragments that is characteristic of RPE (Finnemann et al., 1997a) (Figures 3A–D) and secreted high levels of PEDF (Figure 3E). Using flow cytometry for CRALBP (Hill et al., 2024), the RPE were found to be 98.93% ± 0.74% pure with OCT3/4 expression <1.3 ± 1.6% (mean ± SD, *n* = 4) (Supplementary Figure 1). Residual iPSCs were assayed to <1 in 10,000 cells based on qPCR analysis of pluripotency markers ZSCAN10 and LIN28a (Hill et al., 2024) (Supplementary Figure 2).

Following seeding of RPE on the fibrin hydrogel, cells could be seen to adhere to the region of the top plate containing the gel. By 30 days after seeding, RPE cells could be seen as a pigmented layer of cells sitting atop the fibrin gel (Figures 2A–C,F). Inspection using a stereomicroscope demonstrated that the cells were uniformly distributed and pigmented across the gel (Figure 2B). Under higher magnification using a compound microscope, the cells were found to exhibit a cobblestone-like appearance (Figure 2C). While RPE cells often grew on the polycarbonate sides of the top plate (Figures 2A,B), they adhered poorly and often came off during feeding.

Between 30 and 60 days after seeding we used a custom oval punch (Figures 2D,E) to cut from 60–80 individual doses of ∼1.5 × 5.0 × 0.2 mm from each blank (Figures 2F–I). Each dose contains ∼30,000 cells. Visual inspection of individual doses demonstrated that the RPE were present on a single surface of the gel (Figures 2H–J) and did not appear to penetrate the gel (Figure 2I). It should be noted that handling the gels with forceps did cause damage to the RPE monolayer (left side of Figure 2H). As such, we avoid handling with forceps by using pipettes and pushing devices that did not damage the monolayer during manufacturing. Using OCT, we found that the thickness of the gel did not change during cell culture, and we could clearly distinguish the RPE from the gel based on differences in reflectivity (Figure 2J).

**FIGURE 4:**
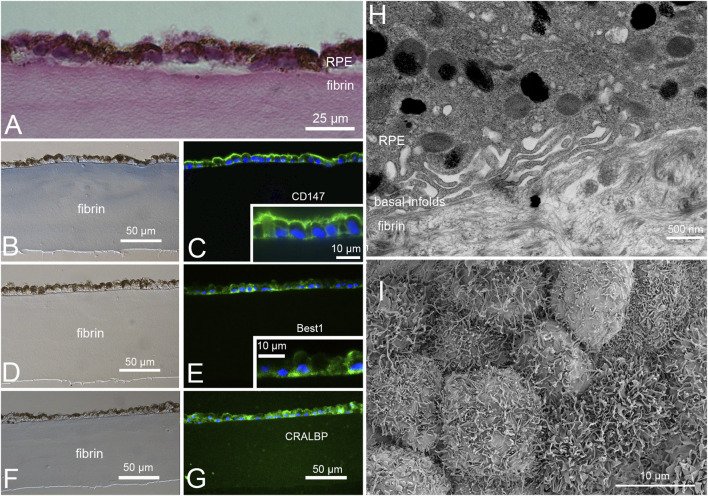
Characterization of iPSC-RPE grown on fibrin gels. Examination of an H&E-stained section of RPE grown on a fibrin hydrogel **(A)** shows that they form a monolayer that sits atop the gel and does not invade it. Immunofluorescence staining **(C,E,G)** and corresponding DIC photomicrographs **(B,D,F)** of RPE on fibrin hydrogels for RPE phenotypical markers (GREEN in C, E, & G) CD147 **(B,C)**, Best1 **(F,G)**, and CRALBP **(F,G)**. CD147 exhibits apical polarity in RPE *in situ* and suggests the presence of microvilli. Insets in C & E are higher magnification images demonstrating apical polarity of CD147 and basal polarity of BEST1. TEM **(H)** shows that the RPE on fibrin gels make basal infoldings but do not invade the gel. SEM imaging **(I)** confirms that the RPE produce abundant microvilli. Nuclei are stained with DAPI (BLUE) in **(C,E,G)**.

H&E staining (Figure 4A) and DIC imaging (Figures 4A,B,D,F) of paraffin embedded sections of RPE on fibrin hydrogels again showed that the thickness of the hydrogel appeared unaltered, that the RPE cells were present as a monolayer of pigmented cells on one surface of the gel, and that they did not penetrate the gel (Figure 4A). TEM found that the cells did not substantially alter the fibrous structure of the gel (Figure 4H) and that the iPSC-derived RPE had numerous basal infolds typical of RPE *in situ* (Figure 4I). Immunofluorescence staining showed that the cells retained expression and proper localization of select RPE phenotype markers; Tra-1-85 (CD147/EMMPRIN) (Figures 4B,C), Best1 (Figures 4D,E), and CRALBP (Figures 4F,G). The Tra-1-85 antibody recognizes human CD147/EMMPRIN, which is expressed uniquely by RPE cells in the eye (Finnemann et al., 1997b; Marmorstein et al., 1996; Marmorstein et al., 1998). Tra-1-85 staining of RPE-fibrin demonstrated the typical polarized distribution of CD147/EMMPRIN to the apical plasma membrane of RPE (Marmorstein et al., 1996; Marmorstein et al., 1998), and suggested that the cells had extensive microvilli (Figure 4C). Microvilli were confirmed by SEM (Figure 4I). Best1 exhibited basolateral polarity (Marmorstein et al., 2000). CRALBP is localized to intracellular compartments (Saari et al., 1984).

**FIGURE 5:**
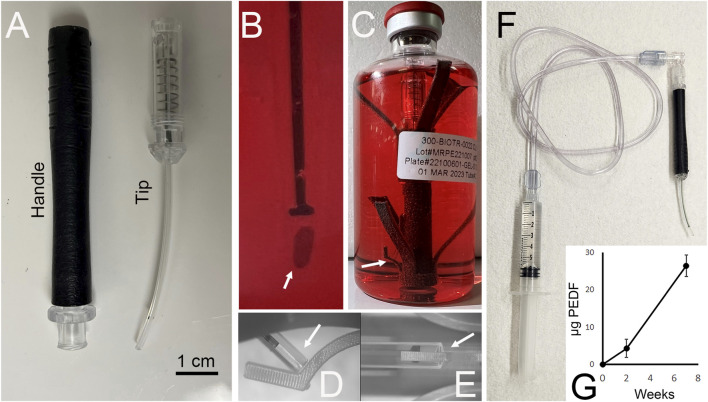
Storage of RPE-fibrin doses. We designed a surgical instrument for storage and transplantation of RPE-fibrin. The inserter is composed of two parts: **(A)** a handle and a tip that screws into the handle. Doses are loaded into the tip **(B)**, and the tip is stored in a crimp sealed R100 vial in a custom clip **(C)**. The clip is designed to prevent the dose (indicated by white arrows in **(B–E)**) from falling out of the tip during storage yet to allow contact between the dose and storage medium. Points of contact between the tip and clip are shown in **(D,E)**. For surgical insertion, the handle is screwed to the tip and connected to a syringe *via* a length of tubing **(F)**. Pressing the syringe plunger results in ejection of the dose from the tip. Single doses remain viable for transplant in the R100 vial and secrete PEDF at 37 °C in the storage medium **(G)** for at least 7 weeks.

Individual doses were placed within the tip of a custom surgical inserter tool (Figures 5A,F). The tool is composed of two main pieces: a tip and a handle (Figure 5A). Both were designed to be for single use and were modified from previously described prototypes (Gandhi et al., 2020; Scruggs et al., 2025; Mano et al., 2022) as follows. The tip was designed to be consistent in diameter along its entire length to accommodate our findings on pressure maintenance during injection as described in Mano et al. (2022). By extending the tip width, a functional seal could be maintained during surgery that maintains intraocular pressure. The wire plunger used in early prototypes was replaced with a laser cut nitinol plunger coated with PTFE. Nitinol was chosen over a stiffer material like stainless steel as it allows some flexibility in the tip while still maintaining shape memory. The PTFE coating promotes a smooth motion of the plunger during insertion and provides protection against corrosion during storage in cell culture medium. This version of the tip was designed to be a storage container as well (Figures 5B,C). Once the dose is loaded in the tip, the tip is packaged in a sterile borosilicate glass R100 vial and filled with 100 mL of RPEM/B27/TXA (Figures 5B–E). The tip is held in place using a custom designed clip that is inserted in advance into the R100 vial (Figures 5C–E). The clip also prevents the dose from falling out during handling while still permitting contact with the cell culture medium in the vial (Figures 5D,E). Vials are crimp sealed and stored at 37 °C until use. Once packaged, the RPE survive and continue to produce PEDF for at least 7 weeks (Figure 5G). Assay of PEDF in the media found an average of 4.38 ± 2.70 µg (mean ± SD, *N* = 6) and 26.45 ± 2.89 µg (average ± SD, *n* = 3) of PEDF accumulated in the media at 2 and 7 weeks, respectively (Figure 5G).

### 2.4 Surgical transplantation

**FIGURE 6:**
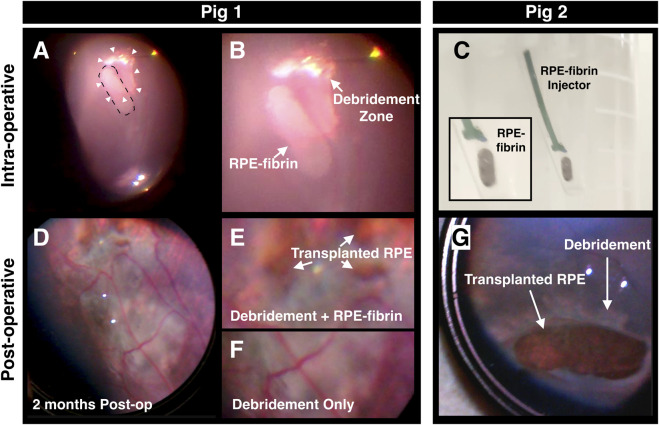
RPE-fibrin transplantation after RPE debridement in a pig model. **(A,B)** Intraoperative fundus images of the posterior retina demonstrating RPE-fibrin implant region (A, dotted line) overlying an area of RPE debridement (A, arrowheads) of pig 1 at a lower magnification **(A)** and a higher magnification **(B)**. **(C)** RPE-fibrin loaded in injection tip immediately before delivery to pig 2. **(D–F)** Fundus images of the posterior retina 2 months post-operatively at lower magnification **(D)** and higher magnification **(E,F)** demonstrating the transplanted RPE **(E)** overlying the RPE debridement zone of pig 1. The fibrin scaffold was no longer present. **(G)** Fundus image at post-operative month 2 demonstrating the transplanted RPE in the subretinal space overlying an area of RPE debridement in pig 2. The fibrin scaffold was no longer present.

For transplantation, the vial is opened and the culture medium decanted. The handle is then used to remove the tip and it is attached to a syringe and tubing (Figure 5F) that have been pre-filled with BSS or sterile water. Exerting pressure with the syringe causes the plunger to deploy the transplant (see Supplementary Video 1). Four domestic pigs underwent RPE debridement and RPE-fibrin transplant surgery as described in Section 4.7 and shown in Supplementary Video 1. The debridement zone, produced by gently scraping the RPE from Bruch’s membrane with a FINESSE® loop ranged from 7–13 mm^2^ (Figures 6A,B). Following debridement, a 3.6 mm sclerotomy was made with an MVR blade. Argon laser was then applied to cauterize the choroid prior to entering the globe with a keratome blade. The transplant inserter was inserted through the sclerotomy, aligned with the retinotomy produced for debridement, and the transplant ejected into the subretinal space (Figures 6A,B) over the debridement zone. Following closure of the sclerotomy a fluid-air, and air-gas [Sulfur hexafluoride (SF6)] exchange was performed, and an intravitreal dexamethasone implant was placed in the remaining peripheral vitreous for immunosuppression.

### 2.5 Post-operative findings

**FIGURE 7:**
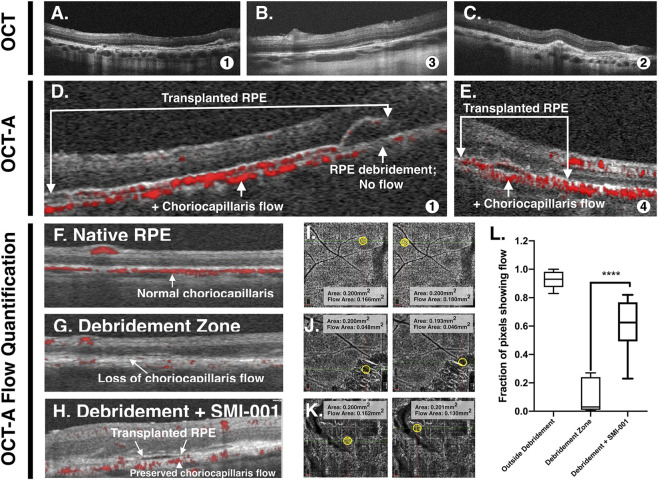
Visualization and quantification of choriocapillaris flow after RPE debridement and transplantation. **(A–C)** Cross-sectional OCT B-scans from three pig eyes showing the RPE transplant **(A)** 1 Month, **(B)** 1 Month, **(C)** 2 Weeks postoperatively in pig 1 **(A)**, pig 3 **(B)**, and pig 2 **(C)**. **(D)** Corresponding OCT-A shows preserved choriocapillaris flow (RED) in transplanted RPE zone compared to adjacent RPE debridement zone with deficit of choriocapillaris flow in pig 1. **(E)** In pig 4, transplanted RPE is associated with preserved choriocapillaris flow (RED) throughout the transplanted region. **(F–H)** Cross-sectional OCT-A B-scans with flow overlay showing: **(F)** native RPE with intact choriocapillaris flow outside the debridement zone; **(G)** debridement zone showing significant loss of choriocapillaris flow; and **(H)** transplanted RPE (Debridement + RPE-fibrin), where choriocapillaris flow is partially preserved. **(I–K)** Corresponding *En face* OCT-A images of the choriocapillaris layer with regions of interest (yellow circles) showing quantitative flow measurements: **(I)** outside debridement zone (normal flow); **(J)** debridement zone (marked flow loss); and **(K)** debridement + RPE-fibrin (preserved flow). Flow areas (mm^2^) are provided for each representative image; these were calculated within a pre-defined 0.5 mm diameter (∼0.2 mm^2^) using Optovue flow software. **(L)** Quantification of flow signal as the fraction of pixels showing flow in each region at 1-month post-op reveals significant reduction in flow in the debridement zone and partial recovery following RPE-fibrin treatment. Box plot shows median ± interquartile range; ****p < 0.0001 by statistical comparison.

**FIGURE 8:**
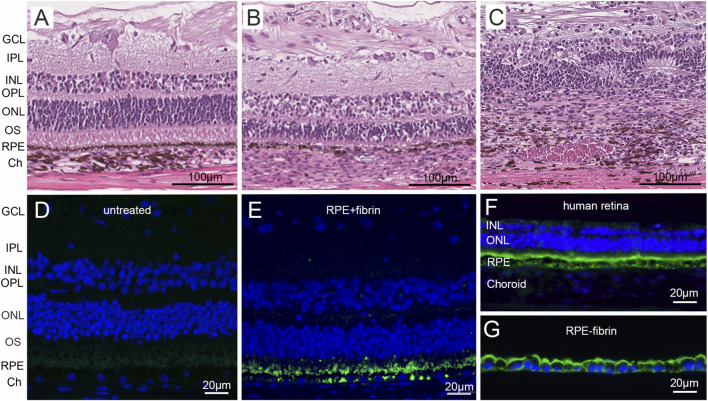
Histology following RPE debridement and RPE-fibrin transplantation. H&E-stained sections from a control region **(A)**, debridement zone containing RPE transplant **(B)**, and debridement zone **(C)** of Pig 2 sacrificed 2 months after receiving the RPE + fibrin transplant. Transplanted RPE in B exhibit mild depigmentation but are in contact with photoreceptor outer segments (OS) which are shortened relative to control regions. There is also thickening of choroid (Ch), loss of choroidal pigmentation, and cellular infiltration. The debridement zone **(C)** exhibits significant disruption of retinal layering with tubulation of surviving photoreceptors, choroidal thickening, and absence of RPE. The presence of transplanted RPE was verified by staining for the human specific RPE antibody Tra-1-85 (Green in **D–G**) which recognizes CD147. Note Tra-1-85 does not stain RPE in the control region of the pig eye **(D)** but does stain transplanted cells **(E)**, RPE in a human eye **(F)**, and the RPE component of RPE-fibrin *in vitro* **(G)**. GCL, ganglion cell layer; IPL, inner plexiform layer; INL, inner nuclear layer; OPL, outer plexiform layer; ONL, outer nuclear layer; OS, outer segment; Ch, choroid. Nuclei are stained in **(D–G)** with DAPI (Blue).

Fundus exam including color fundus photos (Figures 6A,B,D–G), OCT (Figures 7A–C), and OCT-A (Figures 7D–K) were performed at 2 weeks, 1 month, and 2 months post-operatively (Table 1). By week 2, the gas bubble had dissipated in all pigs. Pigs 1 and 3 (Table 1) had vitreous hemorrhage at week 2 which prevented imaging but was cleared by 1 month. In pig 3 the vitreous hemorrhage obscured our view of the retina at 2 weeks. For Pigs 1, 2, and 4 (Table 1) the retina appeared flat over the debridement zone at week 2, though it remained elevated over the transplant site suggesting some residual fibrin scaffold remained. Where we could obtain cross sectional OCT through these areas however, it was noted that the elevated region was filled with hyper-reflective material suggesting inflammation rather than fibrin which is typically hypo-reflective on OCT. The fibrin scaffold in all four pigs was no longer visible and the retina was flattened by 1-month post-transplantation when examined using indirect ophthalmoscopy (Figure 6G) or OCT imaging (Figures 7A–C). Inflammation was observed in every pig receiving a transplant (Figure 8B). This is not unexpected, even with local immunosuppression (Ozurdex) since the RPE component of the transplant is xenogenic. OCT images outside the debridement zone showed normal retinal layers, and corresponding OCT-A flow overlay demonstrated normal flow signal throughout the CC. Regions in which the RPE were debrided exhibited GA and disciform scar in all four pigs after as little as 1 month with complete RPE and outer retinal atrophy (cRORA), pachyvessels, subretinal hyperreflective material (SRHRM), and inner choroidal thinning as well as other features that we have previously described in this model (Iezzi et al., 2024). Within the debridement zone, areas receiving RPE-fibrin transplants could be readily identified post-operatively by indirect fundoscopy as isolated islands of pigment within the cloudy white zone of debridement (Figures 6D–G). In transplant zones, there was no GA, disciform scarring, or cRORA observed (Figures 7A–C), though, similar to the debridement zone, there was SRHRM in all four pigs and pachyvessels in three (Table 1).

**TABLE 1:** OCT and OCT-A findings at time of sacrifice.

​	Pig 1 (22P010)		Pig 2 (22P972)		Pig 3 (22P032)		Pig 4 (22P979)	
Sacrifice	2 months post-op		2 months post-op		1 month post-op		1 month post-op	
iPSC_RPE lot#	MRPE221007		MRPE220828		MRPE221007		MRPE220828	

Findings	Debride zone	Transplant zone	Debride zone	Transplant zone	Debride zone	Transplant zone	Debride zone	Transplant zone
Disciform scar	**+**	**−**	**+**	**−**	**+**	**−**	**+**	**−**
GA	**+**	**−**	**+**	**−**	**+**	**−**	**+**	**−**
CNV type 1	**−**	**−**	**−**	**−**	**−**	**−**	**−**	**−**
CNV type 2	**−**	**−**	**−**	**−**	**−**	**−**	**−**	**−**
cRORA	**+**	**−**	**+**	**−**	**+**	**−**	**+**	**−**
iRORA	**−**	**−**	**−**	**−**	**−**	**−**	**−**	**−**
ORT	±	**−**	**−**	**−**	±	**−**	±	**−**
Pachyvessels	**+**	**+**	**+**	**+**	**+**	**+**	**+**	**−**
Thinning of inner choroid	**+**	**−**	**+**	**−**	**+**	**−**	**+**	**−**
Compl. Choroidal atrophy	**−**	**−**	**−**	**−**	**−**	**−**	**−**	**−**
ORAOS	**+**	**−**	**+**	**−**	**+**	**−**	**+**	**−**
Inner retinal atrophy	**+**	**−**	**−**	**−**	**−**	**−**	**−**	**−**
SRHRM	**+**	**+**	**+**	**+**	**+**	**+**	**+**	**+**
Pigment migration	**+**	**+**	**+**	**+**	**+**	**+**	**+**	**+**
Hypertransmission	**+**	**+**	**+**	**+**	**+**	**+**	**+**	**+**

GA, geographic atrophy; cRORA, complete loss of RPE, and outer retinal layers; iRORA, incomplete loss of RPE, and outer retinal layers; ORT, outer retinal tubulation; ORAOS, outer retinal atrophy overlying scar; SRHRM, subretinal hyperreflective material.

Analysis of *en face* OCT images was performed to compare the area covered by RPE-fibrin vs area debrided at 1-month post-op (Table 2). Since the debridement zone was created mechanically, there was some variability in the total area debrided. The area covered by transplanted RPE at 1 month averaged 4.41 ± 1.44 mm^2^. The surface area of the RPE monolayer on an RPE-fibrin implant ranged between 6.31 and 6.42 mm^2^. The difference in surface is consistent with some loss of cells due to contraction of the RPE monolayer post-implantation, as well as loss of cells due to immune rejection. Segmentation analysis of *en face* OCT-A images was performed to compare CC flow outside the debridement zone to zones of RPE debridement and debridement + RPE-fibrin (Figures 7F–L; Table 3). Consistent with our prior data (Iezzi et al., 2024), CC flow was significantly (p < 0.0001) reduced in the debridement zone compared to untreated regions outside of the debridement zone (Figure 7L). Zones receiving RPE-fibrin transplants also demonstrated significantly (p < 0.0001) less CC flow than regions outside the debridement zone (Figures 7F,G,L). However, RPE-fibrin transplant zones exhibited significantly (p < 0.0001) more CC flow than adjacent debridement zones (Figures 7D,G,H,J–L).

**TABLE 2:** Area (mm^2^) covered by RPE transplant and debridement at 1-month post-op.

Pig	RPE-fibrin^a^ ^,^ ^b^	Debridement only	RPE-fibrin^a^ + debridement
1	2.68	4.39	7.06
2	6.00	8.66	14.66
3	3.91	3.99	7.91
4	5.05	7.87	12.93
Mean ± SD	4.41 ± 1.44	6.23 ± 2.38	10.64 ± 3.76

^a^ Fibrin scaffold was no longer present at 2 weeks.

^b^ Area of RPE-fibrin prior to transplant ranges from 6.31 to 6.42 mm2.

**TABLE 3:** Fractional CC flow/mm^2^.

Pig	Outside debridement (untreated)	Debridement only	RPE-fibrin + debridement
1	0.89 ± 0.03	0.22 ± 0.07	0.74 ± 0.05
2	0.81 ± 0.04	0.22 ± 0.04	0.71 ± 0.11
3	0.86 ± 0.01	0.22 ± 0.07	0.79 ± 0.04
4	0.87 ± 0.04	0.29 ± 0.08	0.66 ± 0.11
Mean ± SD	0.85 ± 0.04	0.24 ± 0.07	0.72 ± 0.09

### 2.6 Postmortem findings

Examination of H&E-stained sections confirmed complete degradation of the fibrin scaffold in all four pigs. These sections also confirmed the loss of RPE in the debridement zone (Figure 8C) resulting in GA characterized by loss of photoreceptor outer segments. In addition, there was notable thinning of the outer nuclear layer (ONL), and immune cell infiltrates. In areas where transplanted RPE were present (Figure 8B), there was some thinning of photoreceptor outer segments, but, as observed by OCT, the ONL remained intact and overall retinal layering was preserved. In transplant zones, there were occasional cellular infiltrates that were absent from outside the debridement zone and debridement only zones. In general, the choroid appeared to be less affected (*e.g.,* thicker) under transplants than in the debridement zone, confirming observations by OCT and OCT-A (Figure 7; Table 1). Immunofluorescence staining for human RPE marker CD147 (Figures 8D–G) confirmed the persistence of transplanted human RPE in the transplant zone even at 2 months post-transplantation (Figure 8E). It should be noted that while pig RPE do express CD147, the Tra-1-85 antibody is specific for human CD147 (Figure 8D). Staining similar to that observed in human retina (Figure 8F) and RPE-fibrin *in vitro* (Figure 8G) was observed in the zones receiving RPE-fibrin transplants (Figure 8E).

## 3 Discussion

Despite the introduction of drugs that inhibit angiopoietin-2, and various complements, GA due to AMD and IRDs remains a significant clinical problem. AMD and many IRDs are due to RPE dysfunction (Fleckenstein et al., 2018; Grenell et al., 2024; Ng et al., 2024; Marmorstein et al., 2000; Marmorstein et al., 2018; Marmorstein et al., 2002). Thus, RPE transplantation seems a logical approach to prevent, halt, or reverse vision loss in these diseases. While RPE transplants have been performed in human clinical trials, the approach must be refined and validated if it is to become the standard of care (Algvere et al., 1994; Algvere et al., 1997; Rao et al., 2025; da Cruz et al., 2018; Mandai et al., 2017; Kashani et al., 2018). Here, we investigated the use of fibrin hydrogels as a scaffold for iPSC-derived RPE. RPE-fibrin combines an allogeneic iPSC-derived RPE monolayer with a high concentration fibrin hydrogel as a scaffold for growth and surgical transplantation (Gandhi et al., 2019; Gandhi et al., 2020; Gandhi et al., 2018; Gandhi et al., 2021). We find that the fibrin hydrogel scaffold used in RPE-fibrin provides a readily degradable scaffold for long-term growth and delivery of a flat monolayer of RPE cells to the subretinal space.

The fabrication of high concentration fibrin hydrogels with control of shape was a difficult challenge. The initial gelation of fibrin at fibrinogen concentrations of ≥30 mg/mL occurs in less than 1–2 s. We previously made the observation that fibrin gelation is slowed by the addition of select azo dyes like trypan blue (Gandhi et al., 2021). This slowing is greater than that observed through changes in pH or temperature (Edsall and Lever, 1951; Gissel et al., 2016; Kaibara and Fukada, 1977) and slows initial gelation sufficient to permit injection into a mold (Figure 1). The gels have a Young’s modulus (0.053 ± 0.01 MPa, mean ± SD) near that of the retina (0.02 MPa) (Ferrara et al., 2021). Thus, they can be inserted into the subretinal space without causing damage to adjacent tissues. Based on our observations (Figures 6–8), the gels are degraded in less than 1 month when inserted together with RPE cells into a pig model of GA that we recently developed (Iezzi et al., 2024). Following degradation of the scaffold, the remaining RPE monolayer settles flat on Bruch’s membrane and establishes contact with the overlying retinal photoreceptors (Figures 7, 8).

Rapid, but controlled, degradation of the fibrin hydrogel is an important property of the RPE-fibrin transplant. In our prior studies using fibrin hydrogels placed in the subretinal space of healthy pig eyes, we found that the scaffold degraded in a period of 8–10 weeks (Gandhi et al., 2020). This was accelerated when the endogenous RPE were debrided resulting in complete degradation within 1 month (Gandhi et al., 2020), similar to the present study. The addition of RPE in the present study, however, interfered with our ability to perform OCT imaging at 2 weeks, presumably due to the immune response to the xenograft despite immunosuppression (Ozurdex implant) administered to every pig at the time of surgery. Prior to transplantation surgery, the RPE-fibrin implant is stored in a medium that contains the antifibrinolytic compound TXA. In the absence of TXA or other antifibrinolytics, the RPE monolayer will degrade the fibrin scaffold (Gandhi et al., 2019; Gandhi et al., 2018). In contrast to aprotinin, TXA is a remarkably stable compound that washes out of the hydrogel readily. This was the basis of our preference for TXA over aprotinin. The ability to inhibit degradation of the fibrin hydrogel during growth and storage of RPE cells, but to easily wash out upon placement in the eye, is a critical property of RPE-fibrin. This property confers the potential advantage of longer storage times over other materials such as PLGA or PDLLA scaffolds (Sharma et al., 2019; Popelka et al., 2015). This property is also critical because it permits storage during final differentiation on the gel blank and lot release testing following packaging of doses. Compendial testing for sterility (e.g., USP<71>), for example, requires 2 weeks. Genetic testing, if required, can take even longer.

In the present study, for the first time, we evaluated the potential efficacy of RPE-fibrin in an animal model of advanced AMD (Iezzi et al., 2024). This caused the surgery to be more complex than in our previous studies (Gandhi et al., 2020). As shown in Figures 6–8, the presence of an RPE-fibrin transplant prevented GA and disciform scarring for up to 2 months. Similarly, the RPE-fibrin transplanted cells preserved significant CC flow, which was severely diminished following mechanical debridement of the endogenous RPE. We are not the first to observe this property of RPE transplantation. The first study using autologous iPSC-RPE observed a similar phenomenon (Mandai et al., 2017; Takagi et al., 2019; Souied et al., 2017), and Gupta et al. (2025) have recently noted that transplanted RPE salvage the CC as well as photoreceptors in a pig retinal degeneration model. Despite these positive findings, it should be noted that some degree of clinical inflammation was noted for all RPE-fibrin transplants. This was not due to the fibrin scaffold, since our prior studies demonstrated that placing an acellular fibrin scaffold in the subretinal space (Gandhi et al., 2020) or adhering an adeno-associated virus containing fibrin hydrogel to the epiretinal surface did not result in inflammation (Scruggs et al., 2025). Despite this, significant changes in inflammatory conditions exist in the AMD eye that could potentially affect safety. However, fibrin tissue glues have been in use for decades with an excellent safety record (Spotnitz, 2014). In the eye fibrin tissue glue is the most common intraocular bioadhesive used in vitreoretinal surgery and is often applied to deal with retinal breaks and tears (Desai et al., 2024; Hansraj and Narayanan, 2025; Lin et al., 2025; Sharabura et al., 2022). In the present study we attempted to mitigate the immune response to human RPE in the pig by placing a dexamethasone (Ozurdex) implant in each pig at the time of surgery. Despite this, rejection of the cells and localized inflammation occurred in all four pigs and remained a significant limitation of the study.

It is not currently clear whether RPE monolayers are superior to cell suspensions. The use of a scaffold allows for control of RPE orientation/polarity and monolayer placement and prevents folding and clumping of transplanted cells. Whether control of these variables is critical to the success of RPE transplantation is currently unknown. A disadvantage of using a scaffold is that the scaffold adds bulk to the RPE monolayer. Monolayer placement in the subretinal space requires a large sclerotomy and retinotomy for subretinal insertion (da Cruz et al., 2018; Kashani et al., 2018; Lytvynchuk et al., 2022; Gandhi et al., 2020; Fernandes et al., 2017). The retinotomy, typically ∼2 mm long, results in a scotoma. In contrast cell suspensions and thin monolayer strips use standard 23 or 25 gauge vitrectomy ports for instrument insertion and a small (31–42 gauge) cannula for subretinal injection (Schwartz et al., 2015; Rao et al., 2025; Sugita et al., 2020). Though complicated by the same issues as unsupported monolayers, the recent use of unsupported RPE strips avoids the additional sclerotomy and the larger retinotomy needed to deliver a large flat monolayer by allowing for insertion of the RPE monolayer through a standard vitrectomy port (Nishida et al., 2021; Ozaki et al., 2025) with subretinal injection *via* a 38 gauge cannula. Thus, the RPE strips potentially provide the benefits of the simpler surgical approach associated with cell suspensions while potentially retaining greater control over area of treatment. Which approach will ultimately prove superior for RPE transplantation remains to be determined. The results of this study suggest that should RPE monolayers prove advantageous, fibrin hydrogels are a safe and effective scaffold for their transplantation, and potentially transplantation of other retinal cell types.

## 4 Materials and methods

### 4.1 Gel fabrication

Gel fabrication was performed aseptically using a modification of our previous method (Scruggs et al., 2025). Fibrin hydrogels were produced using 4 mL Tisseel tissue glue kits (Baxter, cat# 1504518VP). The fibrinogen was resuspended in sodium citrate prepared from a clinical anti-coagulant solution (Fenwal Pharmaceuticals/NDC#-0942–9504–10) diluted to 0.01M with sterile water for injection (Gibco, cat# A1287301) rather than the included resuspension buffer which contains aprotinin. Thrombin was reconstituted with the thrombin solution from the same Tisseel Kit, and the vials incubated in a 37 °C water bath overnight. The following day, 0.6 mL of sterile tissue culture grade 0.4% trypan blue (Gibco, cat# 15250–061) was added to 2 mL of the resuspended fibrinogen and thrombin solutions. The fibrinogen solution was then drawn into an 11 mL syringe and the thrombin solution into a 1 mL syringe. The syringes were placed in a 11:1 ratio FibriJet Ratio Applicator Assembly. The gelation solution was then dispensed through a FibriJet Blending Connector with Mixer (Nordson Medical) and 18G cannula into a custom mold (Meddux, CO) (Figure 1). Molds were incubated at 37 °C for 3 h to allow gels to cure. Subsequently, molds were opened and top plates containing the polymerized gels were hydrated in sterile phosphate buffered saline (PBS) containing 2.5 mg/mL tranexamic acid (TXA). The resulting gel is ∼15.25 × 58.42 × 0.2 mm (0.7″ x 2.45″ x 0.008″) with a final concentration of >30 mg/mL of fibrin.

### 4.2 Determination of fibrin gel properties

Gel thickness was measured using a Lumedica OQ Labscope optical coherence tomography (OCT) system at points of intersection of a 14 × 5 grid drawn on the top plate as described previously (Scruggs et al., 2025). Young’s modulus was determined at the Mayo Clinic Biomechanics Core Facility using a spherical indenter (radius = 0.25 mm) on a MicroTester G2 (CellScale, Waterloo, ON) as before (Scruggs et al., 2025).

### 4.3 Electron microscopy

Both scanning electron microscopy (SEM) and transmission electron microscopy (TEM) were performed at the Mayo Clinic Microscopy and Cell Analysis Core as previously described (Gandhi et al., 2018; Scruggs et al., 2025; Gandhi et al., 2021) using samples fixed in Trumps fixative at 4 °C and embedded in plastic resin for TEM. 100 nm thin sections were viewed using a JEOL 1400 microscope (JEOL; Peabody, MA). SEM samples were viewed and imaged using a Hitachi S-4700 cold field emission scanning electron microscope.

### 4.4 Cell culture and differentiation of RPE

The iPSC line 300-BIOTR-0022 clone 1 (22/1) was produced in compliance with cGMPs from fibroblasts obtained from a 25-year-old Caucasian female donor who met donor eligibility criteria (21 CFR 1271) (Marmorstein et al., 2023). 22/1 cells were maintained and differentiated to RPE at LAgen Laboratories (Rochester, MN) as previously described (Hill et al., 2024). All procedures were carried out aseptically by teams of two or more trained operators in Class 2A biosafety cabinets in an ISO 7 cleanroom. No antibiotics or antimycotics were used in cell culture. All reagents used were produced using GMPs with the exception of Accumax (Innovative Cell Technologies, cat# AM105) and were xenofree with the exception of mTeSR1 (Stem Cell Technologies, cat# 85850), which contains bovine serum albumin from BSE-free herds and is tested for bovine adventitious viruses. A Master Cell Bank (MCB) of iPSC line 300-BIOTR-0022 clone 1 was produced with 0.5 mL of cells cryopreserved in StemCell Banker (DMSO free, GMP grade, AMSBIO, cat# 13926) at 2 × 10^6^ cells/vial. The MCB tested sterile (USP<71>, Eurofins), *mycoplasma* free (PCR, LabCorp), and was free of adventitious viruses (Eurofins). For this study, two lots of RPE cells were produced each from an independent vial of cells. These were the same lots used in Hill et al. (2024). In brief, for RPE production a vial of iPSCs containing 2 × 10^6^ iPSCs was thawed and seeded into a T25 flask coated with Synthemax-2 SC (Corning, cat# 3535). Cells were fed daily with mTeSR1 and grown to confluence in a 37 °C 95% air/5% CO_2_ incubator. Upon achieving confluence, media was changed to CTS-Knock-Out DMEM (GIBCO, cat#) containing 15% (v/v) CTS-Knock-Out SR xenofree medium (GIBCO, cat# 12618013), 1% (v/v) nonessential amino acids (GIBCO, cat# 11140050), 1% (v/v) GlutaMAX (GIBCO, cat# 35050061), and 0.1 mM 2-mercaptoethanol (GIBCO, cat# 21985023) and fed daily. After 49 days, cells were passaged using Accumax, filtered through a 40 µm nylon mesh (Corning, cat# 431750), re-suspended in RPEM/B27 [RPEM (LAgen Laboratories, see Supplementary Table 1) containing 2% CTS-B27 Xenofree supplement (GIBCO, cat# A1486701)] and seeded at 1 × 10^7^ cells in Synthemax-2 SC coated T25 flasks. Cells were fed every other day for 30 days and then passaged again, as described above, prior to being seeded on fibrin gels at 1 × 10^7^ cells/gel blank in RPEM/B27 containing 2.5 mg/mL tranexamic acid [(TXA), Provepharm, NDC# 81284–611–00] (RPEM/B27/TXA). Prior to seeding with iPSC-RPE, gel blanks were washed three times for 20 min in RPEM/B27/TXA. The gel was placed in a 95% air/5% CO_2_ incubator at 37 °C and cells were fed with RPEM/B27/TXA every 2 days for 30–60 days after which individual doses were prepared and packaged as indicated below.

### 4.5 Transplantation device and storage system

A transplantation device that also served for safe storage of the RPE/fibrin gel was developed with assistance from Meddux Corp (CO) based on the prototypes described in Gandhi et al. (2020) and Mano et al. (2022). The current device consists of a disposable handle and tip assembly (Figure 5). The reusable handle has a pneumatic actuator, luer-lok connector, and threads for the disposable tip assembly. The disposable tip assembly consists of a spring-guided pin attached to a polytetrafluoroethylene (PTFE) coated nitinol plunger that is housed within a plastic tube shaped to fit the nitinol plunger. The RPE/fibrin hydrogel transplant is punched from the blank using a custom machined stainless steel punch after 30–60 days of culture. The resultant 1.5 × 5.0 × 0.2 mm oval “doses” were then loaded into tip. Following visual inspection for defects in the cell monolayer and to insure orientation of the monolayer, tips were placed in a custom clip in an R100 vial filled with 100 mL of RPEM/B27/TXA and crimp sealed. The packaged transplants were stored at 37 °C until used. During surgical transplantation the R100 vial was opened using a de-crimping tool, the media decanted, and the inserter handle screwed onto the tip to remove it from the vial. Once the handle and tip were ready, the handle was connected *via* a Luer fitting to a plastic tube and 6cc syringe filled with balanced salt solution. To deploy the transplant from the housing, pressure is applied *via* syringe to the pneumatic actuator causing the transplant to be expelled.

### 4.6 Flow cytometry

Flow cytometry was performed as described previously (Hill et al., 2024) for CRALBP with mouse anti-CRALBP monoclonal antibody B2 (NOVUS, Cat# NB100-74392, RRID: AB_1048601) followed by a PE coupled Goat anti-Mouse IgG1 secondary antibody (Invitrogen, cat# P-21129, RRID: AB_1500811). Flow cytometry for OCT3/4 was performed as described in Hill et al. (2024) using Alexa Flour 488 conjugated mouse anti-Oct3/4 (BD Pharmingen, cat# 560253, RRID: AB_1645304). Data were acquired using a BD FACS Aria II (BD Biosciences) and analyzed using Flowjo Software (Tree Star).

### 4.7 QPCR

QPCR was performed using 10 ng of cDNA and TaqMan™ Gene Expression Assays for ZSCAN10 (Hs00262301_m1), LIN28A (Hs04189307_g1), and β-actin (Hs01060665_g1) as described previously (Hill et al., 2024).

### 4.8 Functional assays

Pigment epithelium derived factor (PEDF) was assayed by ELISA, as previously described (Johnson et al., 2015; Johnson et al., 2013) using conditioned medium collected from P0 cells at 49 days and for P1, P2, and RPE-Fibrin at 30 days post-plating. The phagocytosis function of iPSC RPE was determined as before (Muller et al., 2018). In brief, photoreceptor outer segment fragments (POS) were prepared from fresh pig eyes and stored as frozen stock at −80 °C (Parinot et al., 2014). Thawed POS were covalently labeled with Texas Red by incubation with Texas Red™-X Succinimidyl Ester (Thermofisher, cat# T6134) at 30 μg/mL in 0.1M Na-bicarbonate buffer pH 9.5 for 1 h on the day of the experiment. iPSC RPE on glass coverslips were serum starved for 1 h prior to incubation for 5 h with labeled POS at a concentration of ∼10 particles per cell. POS were fed in DMEM alone, or in DMEM supplemented with purified human MFG-E8 (Biotechne cat# 2767-MF) and Protein-S (Biotechne, cat# 9489-PS-100), at 2 μg/mL each in the presence of 1% PBS (as solvent control) or of 1% cilengitide (MilliporeSigma, cat# 188968–51–6) stock solution (100 μM in PBS). Cells were washed three times with PBS, fixed with 4% paraformaldehyde, and counterstained with 4′,6-Diamidino-2-Phenylindole (DAPI) before mounting on microscope slides. Phagocytosed POS were quantified by flatbed fluorescence scanning and visualized on a Leica TSP8 confocal microscopy system, as described previously (Muller et al., 2018). Four independent phagocytosis assays with triplicate samples each were performed and analyzed.

### 4.9 Animals and surgery

All animal procedures were approved by the Institutional Animal Care and Use Committee (IACUC) of Mayo Clinic and conducted in accordance with the ARVO Statement for the Use of Animals in Ophthalmic and Vision Research and National Institutes of Health guidelines. Female domestic pigs (*Sus scrofa* domesticus) at 2 to 3-month of age and weighing between 20 and 35 kg were used for this study. For all animals the right eyes (OD) were designated as the experimental/operative eyes. RPE-fibrin transplantation was performed by two different surgeons (RI, pigs 1-3, BAS pig 4; see Table 1), as described previously (Gandhi et al., 2020) following RPE debridement as before (Iezzi et al., 2024) (see Supplementary Video 1). Two different blanks were used each with a distinct lot of iPSC-RPE as indicated in Table 1. RPE-Fibrin was surgically implanted within 18 days of packaging. All operated eyes received a dexamethasone implant (Ozurdex®, Allergan, NDC No. 0023–3348–07) for immunosuppression which was placed in the remaining peripheral vitreous at the end of the procedure.

### 4.10 Color fundus photography, OCT, & OCT-angiography (OCT-A)

Pigs were anesthetized using isoflurane, as before (Gandhi et al., 2020; Scruggs et al., 2025; Iezzi et al., 2024) for each post-operative examination. Eye drops were instilled for dilation and topical anesthesia as above. Color fundus photos, OCT, and OCT-Angiography (OCT-A) were performed at each exam. Fundus photographs were obtained using a custom-made video indirect ophthalmoscope. Images were processed using Photoshop (Adobe, San Jose, CA). OCT, OCT-A, and infrared SLO were performed using the Optovue Avanti OCT Angiovue System (Visionix; North Lombard, IL).

Quantification of choriocapillaris flow was performed using 6 × 6 mm OCT-A scans. For each pig, five regions outside the RPE debridement zone (native RPE), five regions within the RPE debridement zone, and five regions within the transplant region (RPE debridement + RPE-fibrin) were randomly selected based on high-quality OCT-A image acquisition. Each region was analyzed using Optovue’s flow area analysis software. To standardize quantification across pigs, we used a predefined 0.5 mm diameter measurement circle, corresponding to an approximate area of 0.2 mm^2^. Flow area within each circle was quantified as the fraction of pixels exhibiting flow signal, as determined by the device’s built-in flow detection algorithm within the segmented choriocapillaris slab.

### 4.11 Histology

Pigs were euthanized at 1 or 2 months post-op (Table 1) by rapid intravenous injection of a pentobarbital solution [*FATAL-PLUS*, Vortech (NDC No. 0298–9373–68); 1 mL/per 10 lbs of body weight]. Eyes were enucleated, fixed in Davidson’s fixative, and processed into paraffin, as previously described (Scruggs et al., 2025). iPSC-derived RPE on fibrin gels were fixed in neutral buffered formalin prior to embedding in paraffin. 5 μm sections were cut and either stained with H&E or processed for immunofluorescence as described below. H&E stained sections were scanned using a Leica Aperio® AT2 microscope slide scanner and viewed and images captured using Aperio Imagescope software. Human Eyes were fixed and processed as described in Marmorstein et al. (2002).

RPE monolayers on fibrin hydrogels were fixed in neutral buffered formalin for >24hrs, then processed for paraffin histology. Immunofluorescence was performed, as described previously (Scruggs et al., 2025; Hill et al., 2024), using rabbit polyclonal antibody Pab125 to detect Best1 (Marmorstein et al., 2000), CD147/EMMPRIN using mouse monoclonal antibody Tra-1-85 (R&D Systems, cat# MAB3195, RRID: AB_2066681), or CRALBP using mouse monoclonal antibody B2 (Novus, cat# NB100-74392, RRID: AB_1048601). Nuclei were counterstained using 4′,6-diamidino-2-phenylindole (DAPI). Immunofluorescence and transmitted light images were obtained on a Nikon E600 microscope (Nikon Instruments, Melville, New York, USA) using a CCD camera and Nikon Elements Software.

### 4.1 Statistical methods

Data are presented as mean ± standard deviation (SD) or standard error (SE) as indicated. Statistical significance was determined using the TTEST function in Microsoft Excel, except for phagocytosis assays where statistical significance was determined using one-way ANOVA and Tukey *post hoc* testing (GraphPad Prism).

## Data Availability

The original contributions presented in the study are included in the article/Supplementary Material, further inquiries can be directed to the corresponding author.
