# Supplementary material for: Degradable fibrin hydrogels for transplantation of iPSC-derived retinal pigment epithelial cell monolayers
Source: Front Cell Dev Biol. 2026 Jan 14;13:1739620. doi: 10.3389/fcell.2025.1739620 (PMC12848544; doi:10.3389/fcell.2025.1739620)
Supplement: Supplementary file 1 [file DataSheet1.pdf]

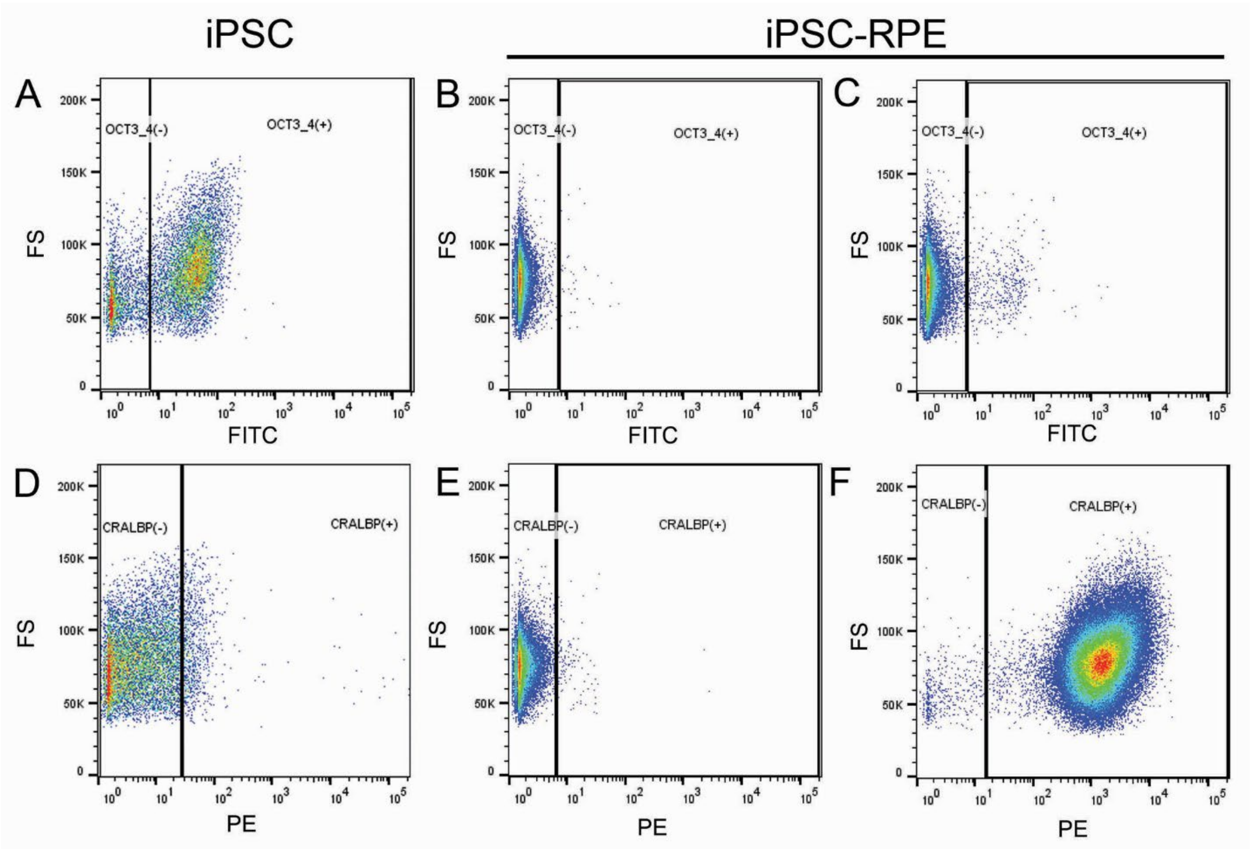

Supplementary Figure 1. Purity of iPSC-derived RPE. Representative flow cytometry data for OCT3/4 (A, B, C) and CRALBP (D, E, F) expression in iPSCs (A, D) and iPSC-RPE (B, C, E, F) by flow cytometry as described in (1). Panel A shows OCT3/4 staining of iPSCs and panel B shows unstained iPSC-RPE used to set gating parameters for OCT3/4 staining of iPSC-RPE in panel C. Panel D shows CRALBP staining of iPSCs and panel E shows unstained iPSC-RPE used to determine gating parameters for CRALBP staining of iPSC-RPE in panel F. FS = forward scatter.

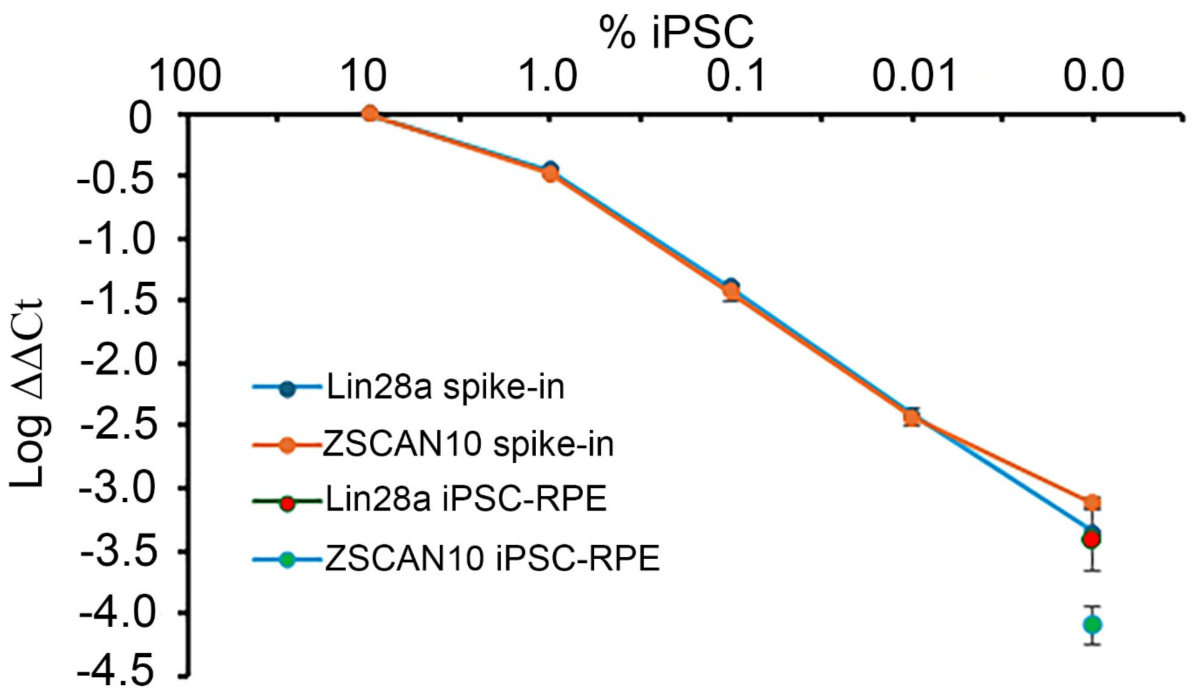

Supplementary Figure 2. Detection of Residual iPSCs in iPSC-derived RPE. Quantitative PCR for Lin28a and ZSCAN10 was used to determine the % of residual iPSCs in RPE derived from the 22/1 iPSC line and grown on fibrin hydrogels as described previously (1). The average ± SE for 4 independent assays for Lin28a (**RED** circle) and ZSCAN10 (**GREEN** circle) is shown compared to data from samples in which Lin28a (**BLUE** circle) and ZSCAN10 (**ORANGE** circle) was assayed at known ratios of iPSCs to RPE. Note that sensitivity is to <1 in 10,000 RPE (0.01%) based on our prior data (1).

Supplementary Table 1. RPEM Formulation*

**Amino Acids**	Concentration (mM)
Glycine	0.31
L-Alanine	0.029999998
L-Arginine hydrochloride	0.578672996
L-Asparagine-H_2O	0.030020001
L-Aspartic acid	0.03
L-Cysteine hydrochloride-H_2O	0.059863632
L-Glutamic Acid	0.03
L-Glutamine	0.3
L-Histidine hydrochloride-H_2O	0.17
L-Isoleucine	0.570228995
L-Leucine	0.59106869
L-Lysine hydrochloride	0.618306005
L-Methionine	0.009060403
L-Phenylalanine	0.289090909
L-Proline	0.09
L-Serine	0.31
L-Threonine	0.588823536
L-Tryptophan	0.057901963
L-Tyrosine disodium salt dihydrate	0.287869942
L-Valine	0.59239316

**Vitamins**	
Biotin	8.97541E-06
Choline chloride	0.05
D-Calcium pantothenate	0.006184486
Folic Acid	0.00723356
Niacinamide	0.023039343
Pyridoxine hydrochloride	0.013812869
Riboflavin	0.000774202

Thiamine hydrochloride	0.008575668
Vitamin B12	0.000309963
i-Inositol	0.058

Inorganic Salts

Calcium Chloride ($CaCl_2$) (anhyd.)	1.35104505
Cupric sulfate ($CuSO_4$-$5H_2O$)	0.000003
Ferric sulfate ($FeSO_4$-$7H_2O$)	0.0009
Magnesium Chloride (anhydrous)	0.18069474
Ferric Nitrate ($Fe(NO_3)3 \cdot 9H_2O$)	0.000173267
Magnesium Sulfate ($MgSO_4$) (anhyd.)	0.56974162
Potassium Chloride (KCl)	4.6277335
Sodium Bicarbonate ($NaHCO_3$)	35.033334
Sodium Chloride (NaCl)	116.5465502
Sodium Phosphate dibasic (Na_2HPO_4) anhydrous	0.93405797
Zinc sulfate ($ZnSO_4$-$7H_2O$)	0.000898958

Other Components

D-Glucose (Dextrose)	20.5033333
Hypoxanthine Na	0.009
Linoleic Acid	9E-05
Lipoic Acid	0.000305825
Phenol Red	0.028852285
Putrescine 2HCl	0.0003
Sodium Pyruvate	0.3
Thymidine	0.000867769

*RPEM was manufactured under cGMPs for LAgen Laboratories LLC by GIBCO at their Grand Island, N.Y. Facility.

References

1. M. Hill, C. Andrews-Pfannkoch, E. Atherton, T. Knudsen, E. Trncic and A. D. Marmorstein: Detection of Residual iPSCs Following Differentiation of iPSC-Derived Retinal Pigment Epithelial Cells. *J Ocul Pharmacol Ther*, 40(10), 680-687 (2024) doi:10.1089/jop.2024.0130
